# Evaluation of fatigue performance of asphalt materials based on their relaxation behavior

**DOI:** 10.1038/s41598-025-85583-1

**Published:** 2025-01-08

**Authors:** Beibei Zhang, Mengqi Jing

**Affiliations:** Shanxi Province Land Engineering Construction Group Co., Ltd, Xian, 710075 China

**Keywords:** Asphalt mastic, Stress relaxation, Relaxation index, Fatigue performance, Fatigue prediction equation, Civil engineering, Materials science

## Abstract

Although the fatigue properties of asphalt materials have been extensively studied, the relationship between the rheological properties and road performance of asphalt mixtures remains underexplored. In this study, we have examined the relaxation properties of asphalt binders through relaxation tests conducted on asphalt and its mastic under different conditions. A repeated stress relaxation-recovery test is designed for assessing both the relaxation and elastic properties, and a set of reasonable test parameters is recommended, thereby establishing a novel test method for measuring the relaxation and elastic behaviors of asphalt. In addition, we have proposed evaluation indexes, such as relaxation time, relaxation rate, and strain recovery rate, to assess the stress relaxation performance and strain recovery ability. It is observed that the relaxation rate and strain recovery rate of the material can be used to characterize the material’s relaxation and elasticity properties, respectively. Thus, the proposed indexes can be used to comprehensively evaluate the viscoelastic performance of the material. The fatigue performance of the selected materials is further examined using the linear amplitude sweep (LAS) test, and the correlation between the relaxation properties and fatigue performance (as indicated by fatigue parameters) is explored. Furthermore, a fatigue performance prediction equation based on the repetitive stress relaxation-recovery test is established. The findings reveal a strong correlation between the relaxation properties and fatigue performance, suggesting that the stress relaxation test can accurately assess the fatigue performance of asphalt materials.

## Introduction

Asphalt is the most widely used pavement material because of its superior viscoelastic properties compared with other road building materials^1^. However, asphalt’s aging performance is limited, making it susceptible to fatigue failure under repeated traffic loads. In the early stages of service, this manifests as a loss of stiffness modulus^2^. As the modulus decreases, the pavement’s performance is inevitably weakened, which further exacerbates the loss of its modulus. This type of fatigue damage plays a decisive role in the lifespan of asphalt pavements^3,4^. Asphalt mixture, the primary component of pavements, plays a crucial role in resisting fatigue damage. Understanding its fatigue resistance is essential for road design, construction, maintenance, and repair^5,6^. Therefore, evaluating the fatigue life of asphalt pavements under specific conditions is the key to guiding and improving the pavement design^7,8^. Hao Peiwen demonstrated that the creep index was more effective in evaluating the low-temperature performance of asphalt mixtures through bending creep tests^9^. Chen Huaxin et al. also used bending creep tests to assess the low-temperature performance of asphalt mixture, finding that this method can effectively evaluate the low-temperature performance of the mixture^10^. Pi Yuhui et al. studied the anti-fatigue properties of asphalt mixture using static load splitting tests, establishing a relationship between the creep failure time and fatigue life. This allowed for an evaluation of the anti-fatigue performance based on splitting creep^11^.

Asphalt mixture is a viscoelastic composite material, and its pavement performance is strongly affected by loading and environmental conditions. By exploring the rheological properties and relaxation behavior of asphalt material and its mortar, we can analyze their impact on the material’s fatigue resistance^12–14^. Previous studies have demonstrated a strong correlation between the high-temperature performance of asphalt materials and their high-temperature creep characteristics. Specifically, the non-recoverable creep compliance and strain recovery rate of asphalt binder can be used to effectively evaluate its high-temperature rutting resistance^15^. The relaxation characteristics of the material are a good indicator of its medium- and low-temperature performance. It has been reported that materials with good low-temperature relaxation performance tend to have better low-temperature cracking resistance^16–18^. Moreover, the fatigue prediction models for asphalt mixture that account for moderate temperature relaxation have shown good reliability^19^.

However, there is still a lack of research on the correlation between the basic viscoelastic rheological properties and the fatigue properties of asphalt materials and their mortar^20,21^. To address this gap, this study investigates the relaxation properties of asphalt and its mortar^22^. The rheological properties of asphalt materials are evaluated using relaxation tests, repeated strain relaxation-recovery tests, and stress relaxation performance index analysis of viscoelastic materials^23,24^. In addition, the linear amplitude sweep (LAS) test is conducted to examine the fatigue properties of the asphalt material^25^, and the viscoelastic continuum damage (VECD) model is used to process the test results^26^. Subsequently, the damage curve and a fatigue prediction equation for the material are obtained. Finally, the correlation between the fatigue properties and viscoelastic indexes of materials is analyzed^27^, establishing a numerical relationship between relaxation properties and fatigue characteristics. This enables the creation of an effective fatigue performance evaluation method based on repeated stress relaxation-recovery tests.

## Materials

### Asphalt and mineral filler

ESSO 50#, 70#, 90# asphalts, along with their corresponding styrene-butadiene-styrene (SBS)-modified asphalt grades, 70# SBS and 90# SBS, were utilized in this study. The main technical indicators of asphalt are listed in Table 1. The results confirm that the tested asphalt binders meet the technical requirements for construction.

**Table 1:** Conventional technical specifications for asphalt.

Index	50#	70#	90#
Penetration 25 ℃, 100 g, 5 s, 0.1 mm	58	74	87
Ductility at 10 ℃ (cm)	28	34	45.7
Softening point (℃)	60.4	49.7	48
Quality loss (%)	0.191	0.019	0.11
Residual penetration ratio at 25 ℃ (%)	76.5	62.3	60.5
Residual ductility at 10 ℃ (cm)	6.3	12.5	16.2

The main technical performance indexes of the mineral powder are listed in Table 2, which are in accordance with the specification requirements.

**Table 2:** Conventional technical specifications of mineral powder filler.

Limestone powder (LS)	
Index	Value
Apparent density	2.703 g/cm^3^
Hydrophilic coefficient	0.631
Moisture content	0.43%
Percentage of particles that can pass through 0.60 mm sieve	100%
Percentage of particles that can pass through 0.15 mm sieve	95.6%
Percentage of particles that can pass through 0.075 mm sieve	78.35%

### Stress relaxation test

#### Preparation of aged bio-asphalt

Firstly, an asphalt specimen with good fluidity was prepared and weighed for casting. According to the requirements of the standard aging test^28^, short-term aging was performed to collect the residue, which was followed by pressure aging to maintain a suitable degree of fluidity in the asphalt specimen. This ensured that the specimen formed a uniform film on the surface of the test plate. The residue was collected after the pressure aging test.

#### Preparation of asphalt slurry

Based on engineering experience, the powder-to-asphalt ratio was set at 1.0. The asphalt specimen was placed in an oven for temperature control to ensure adequate mobility. The dried mineral powder was weighed and then added to asphalt in increments of 10 g each time. A high-speed electric mixer, operating at 1000 rpm, was used for mixing. After each addition of mineral powder, the mixture was uniformly stirred for 10 min. After the last addition, the mixture was stirred for 20 min to ensure complete blending of the mineral powder and asphalt^29,30^. The different types of asphalt slurries are listed in Table 3.

**Table 3:** Types and combinations of asphalt slurries.

Processing method	Asphalt type				
Untreated asphalt	50#	70#	70#SBS	90#	90#SBS
Long-term aging	50#LA	70#LA	70#SBSLA	90#LA	90#SBSLA
Asphalt mastic	50#M	70#M	70#SBSM	90#M	90#SBSM

## Experimental investigation of stress relaxation properties of asphalt materials

### Experimental design

#### Simple stress relaxation test

A dynamic shear rheometer (DSR) was utilized to conduct stress relaxation tests^31^. A cylindrical specimen with a diameter of 8 mm was used as the test specimen. The specimen was placed on the lower disk of the instrument, and the initial gap between the upper and lower disks was set to 2050 μm to remove any excess asphalt. The gap was then adjusted to 2000 μm. Subsequently, the test temperature, load (strain value), and duration were set before starting the experiment.

The control parameters for the relaxation test^32^ were set as follows: loading time of 100 s, test temperatures of 10, 20, and 40 °C, and strain loads of 0.2%, 0.4%, 1%, 5%, and 10%. These strain values were chosen based on the fact that the asphalt and its mastic in the mix typically experience strain loads of less than 10% during service. Prior to data analysis, the relaxation curves were normalized to the initial stress values, with the initial points of the relaxation curves set to unit 1 to maintain the same basic trend.

#### Repeated stress relaxation-recovery test

**Fig. 1:**
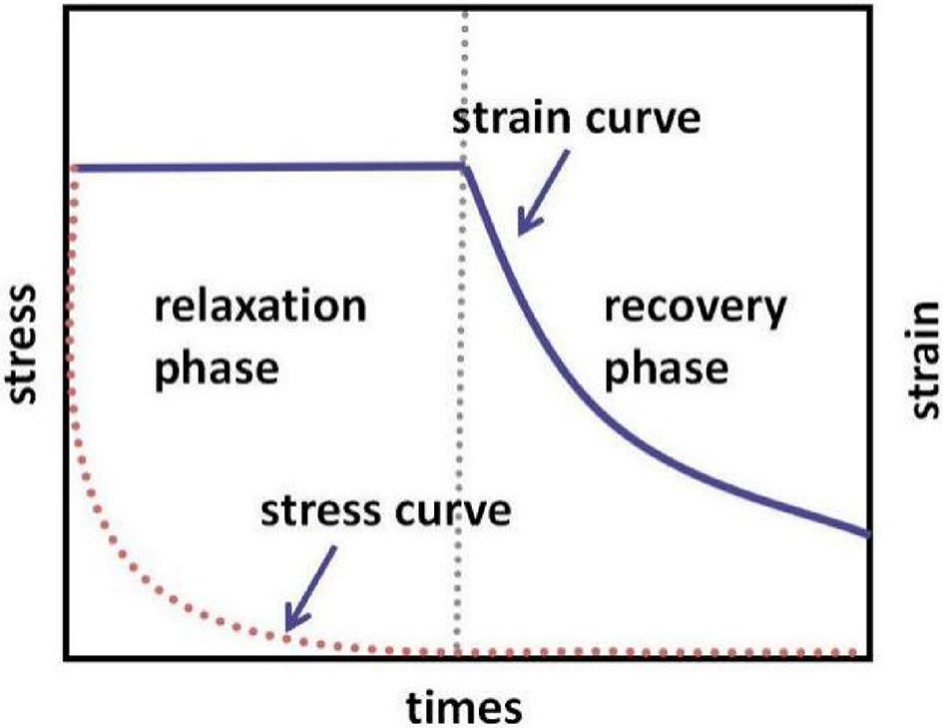
Schematic of the stress vs. strain curve obtained from the stress relaxation-recovery test.

**Fig. 2:**
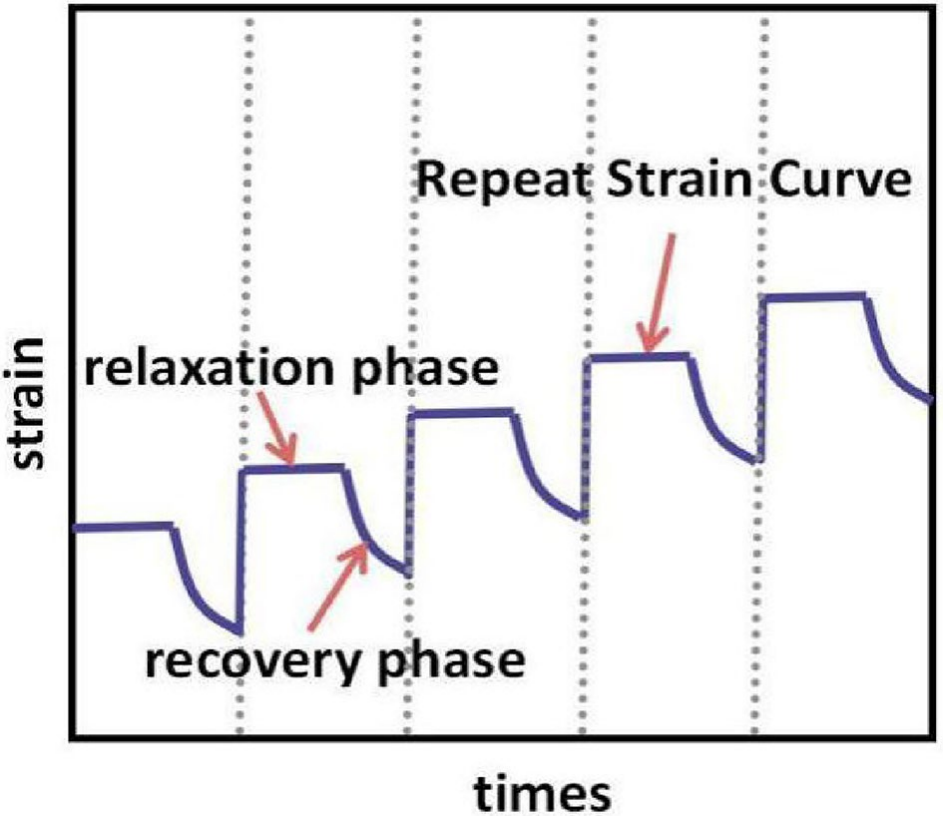
Schematic of the loading time vs. strain curve obtained from the repeated stress relaxation-recovery test.

As a typical viscoelastic material^33^, asphalt experiences fatigue damage under repeated traffic loading, which is accompanied by stress relaxation and damage accumulation. The relaxation properties of the material are intrinsically linked to its fatigue properties. The viscoelastic properties of asphalt materials encompass both stress relaxation and elastic properties, necessitating a test divided into relaxation and recovery phases. Firstly, the relaxation curve is obtained by applying a constant load, allowing the material to undergo stress relaxation. Subsequently, the constant load is removed, enabling the material to recover a certain amount of residual elastic strain, which can then be used to analyze its elastic properties. The specific loading process is illustrated in Fig. 1. To ensure test reliability, the material was subjected to multiple cyclic tests within the same step. As shown in Fig. 2, the cyclic test includes multiple repetitions of the two-stage relaxation-recovery procedure.

#### Fatigue performance analysis based on relaxation test

**Fig. 3:**
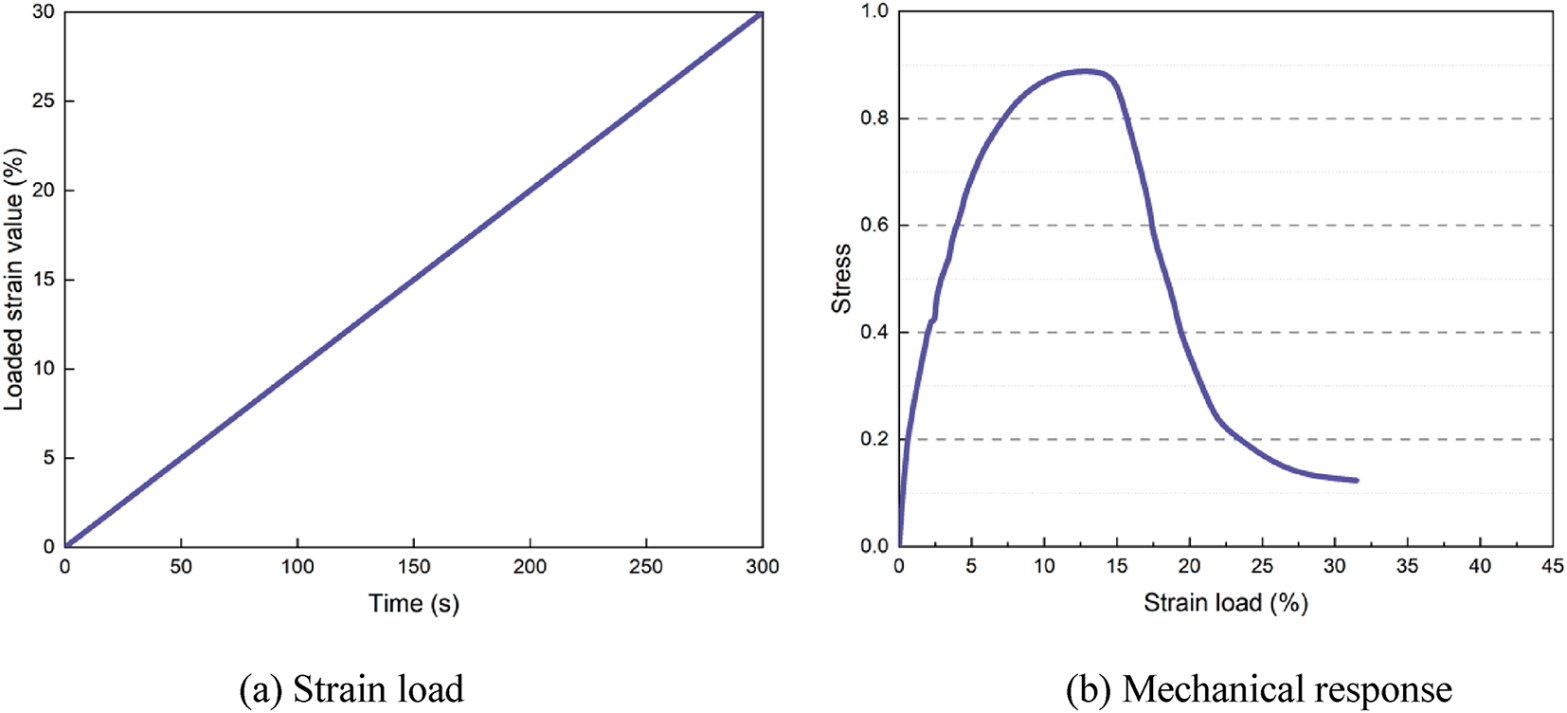
Strain load and stress response obtained from the LAS test.

The LAS test was used to evaluate the fatigue resistance of asphalt binders^34,35^, with long-term aged residual asphalt specimens employed for the analysis. To ensure that the test material was consistent with the repeated stress relaxation-recovery test, the fatigue performance test was conducted at a temperature of 20 °C, with a frequency range of 0.01–50 Hz. The loading frequency was kept constant throughout the test. During the LAS test, the strain load was linearly increased. The variation in the load strain is shown in Fig. 3(a). It can be seen that the strain load range is 0.1–30%, and the loading time is 300 s. The corresponding stress response curve is depicted in Fig. 3(b), which can be used to obtain the fatigue damage curve of the asphalt binder and its mastic.

### Selection of relaxation test parameters

#### Strain load

**Fig. 4:**
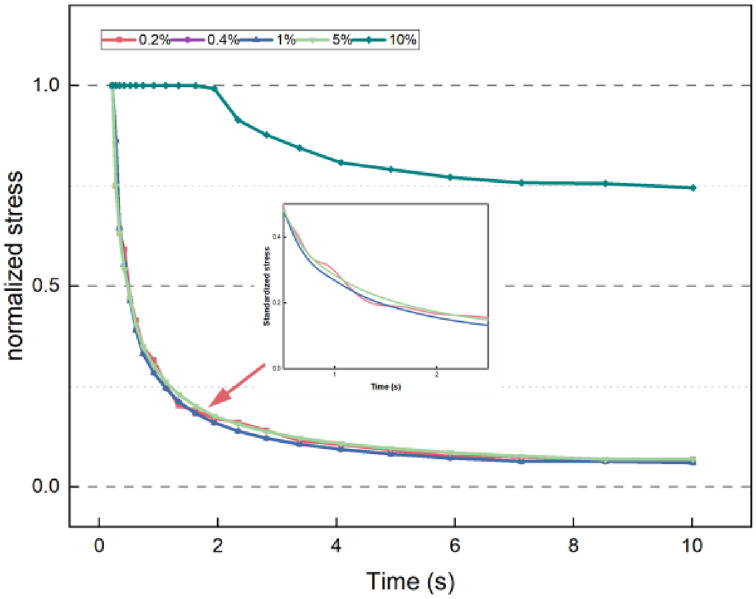
Relaxation curves under five different strain conditions for 90# aged asphalt.

The stress relaxation test was conducted using five strain levels: 0.2%, 0.4%, 1%, 5%, and 10%^28^, as shown in Fig. 4. The stress curve for the 90# base asphalt mastic at a strain load of 10% remains horizontal in the early stages, indicating that a larger strain load may damage the material and yield unreliable test results. The 90# asphalt mastic loaded at 20 °C exhibits large fluctuations in the relaxation curve at 0.2% strain, and similar fluctuations are observed at 0.4% strain. Although the strain load does not significantly affect the overall trend of the relaxation stress curve, the curve is not smooth at lower strain levels. This is attributed to the small internal stress response of the material at low strain loads, which can lead to errors in test control and data acquisition, causing fluctuations. Notably, fracture occurred during the actual experiment at a strain of 5%. Therefore, a strain load of 1% was selected for further analysis.

#### Relaxation time

Simple stress relaxation test.

**Fig. 5:**
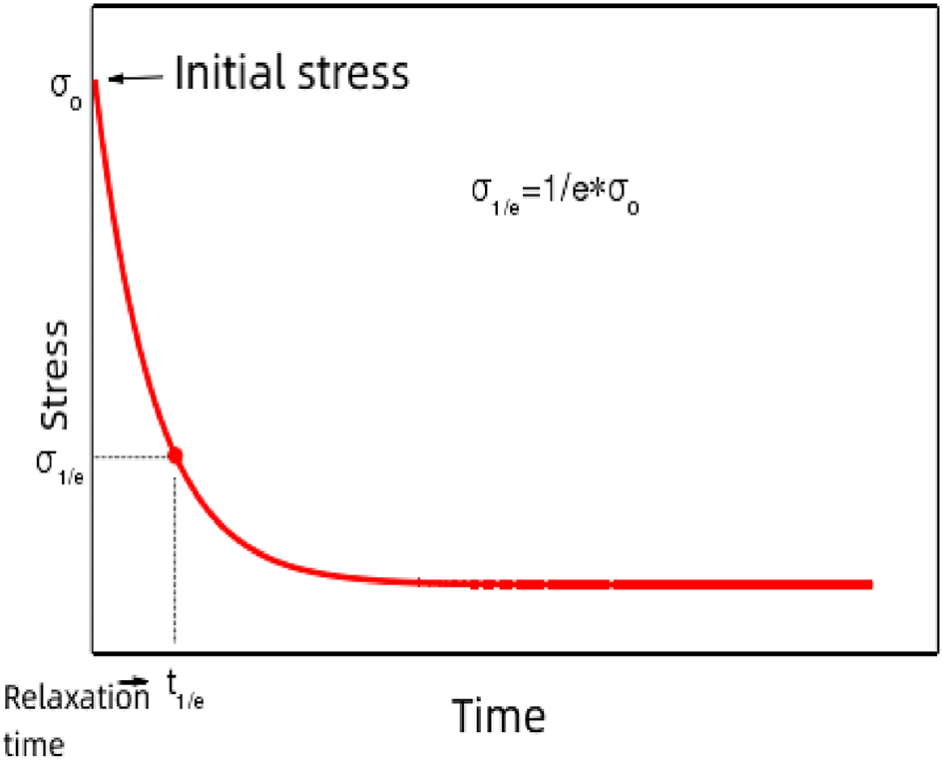
Definition of relaxation time. Stress relaxation completion time of various types of asphalt.

**Fig. 6:**
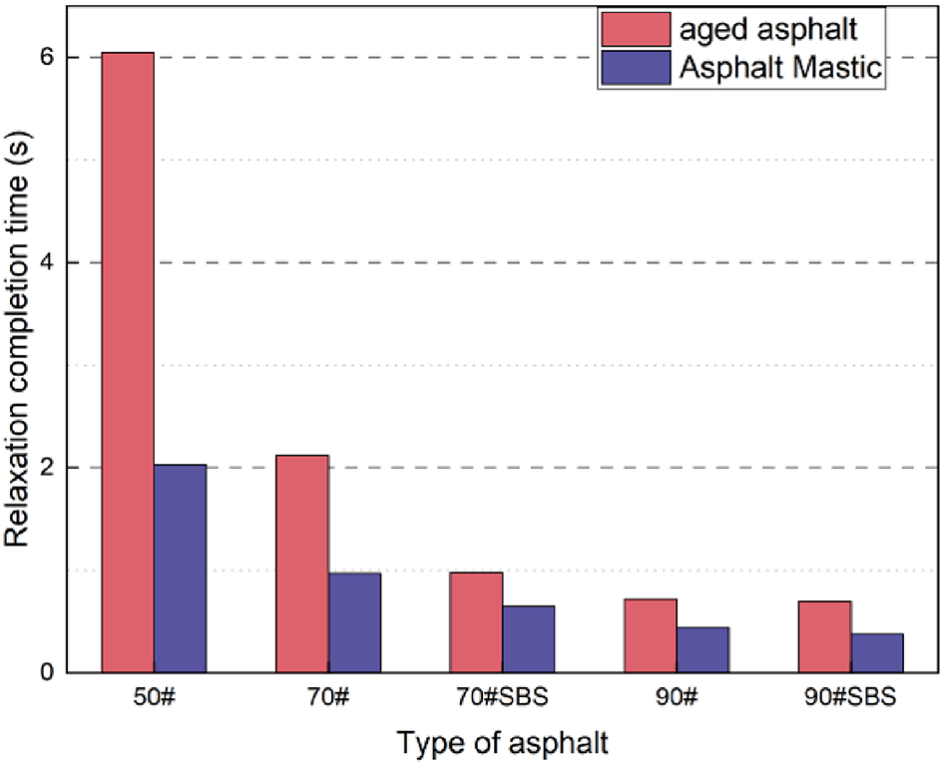
Stress relaxation completion time of various types of asphalt.

The stress relaxation completion time is defined as the moment when the stress reaches 0.1 times the initial stress value in the relaxation curve^37^. As shown in Fig. 5, the stress relaxation process primarily occurs within the relaxation completion time, which verifies the feasibility of the above definition. Figure 6 shows the relaxation completion time for different kinds of asphalt materials. Untreated asphalt is relatively soft, resulting in a very short relaxation completion time. By contrast, high-grade asphalt completes stress relaxation within 0.1 s. Aged asphalt and asphalt slurry exhibit relatively longer relaxation completion times, as illustrated in Fig. 6.

For aged asphalt and asphalt slurry, a correlation exists between the relaxation completion time and asphalt grade: the relaxation completion time decreases as the asphalt grade increases. The addition of a modifier increases the relaxation completion time of asphalt, with pressure aging yielding a more significant improvement than the addition of mineral powder. It can be seen in Fig. 6 that the maximum relaxation completion time for the ten types of asphalt is 6.05 s, while the minimum time is 0.18 s. Therefore, for the subsequent analysis, a relaxation time of 10 s is selected to encompass all asphalt materials tested under the conditions of 20 °C and 1% strain.

(2) Repeated stress relaxation-recovery test.

**Fig. 7:**
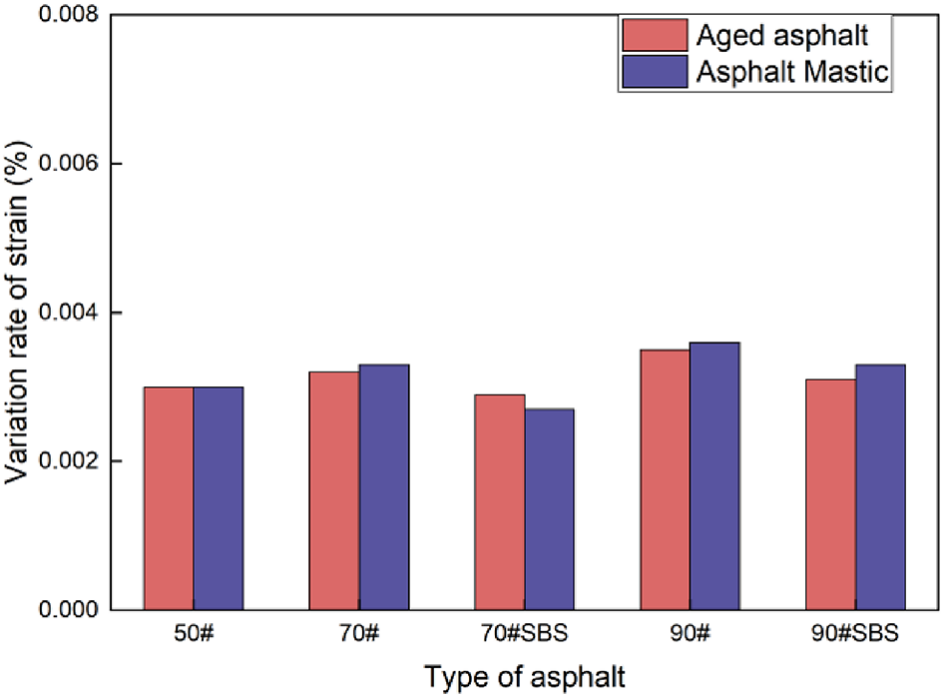
Variation rate of strain at 50 s for various types of asphalt samples in the recovery stage.

In the repeated stress relaxation-recovery test of asphalt materials, the relaxation phase was set to last for 10 s, while the recovery phase lasted for 100 s. Previous studies have indicated that the strain variation rate of the material approaches zero after 100 s of strain recovery. Considering the loading and unloading conditions during actual service, the strain recovery time should be appropriately reduced. Figure 7 displays the strain recovery curve based on the stress relaxation-recovery test conducted for 50 s. As the asphalt grade increases, the strain variation rate consistently decreases. For the same kind of asphalt, the addition of SBS modifier significantly enhances the elastic properties, leading to a notable increase in the strain variation rate at 50 s. The strain variation rate of all the materials at 50 s is approximately 0.002%/s. Therefore, a strain recovery time of 50 s is selected for the subsequent analysis.

## Results and discussion

### Viscoelastic analysis of asphalt materials

**Fig. 8:**
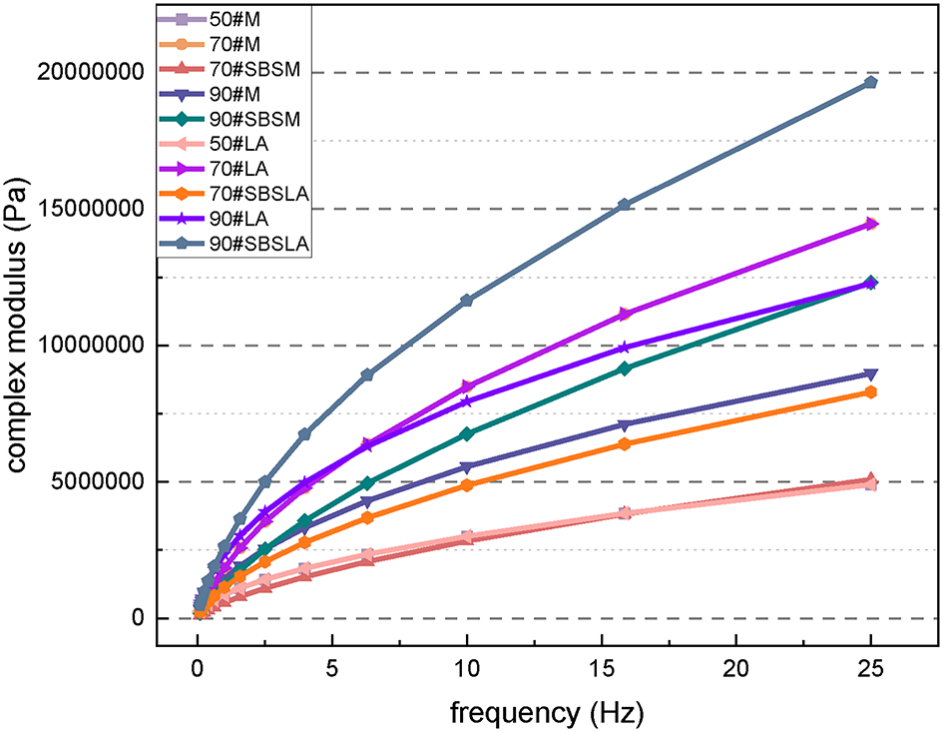
Complex modulus curves for different asphalt materials.

**Fig. 9:**
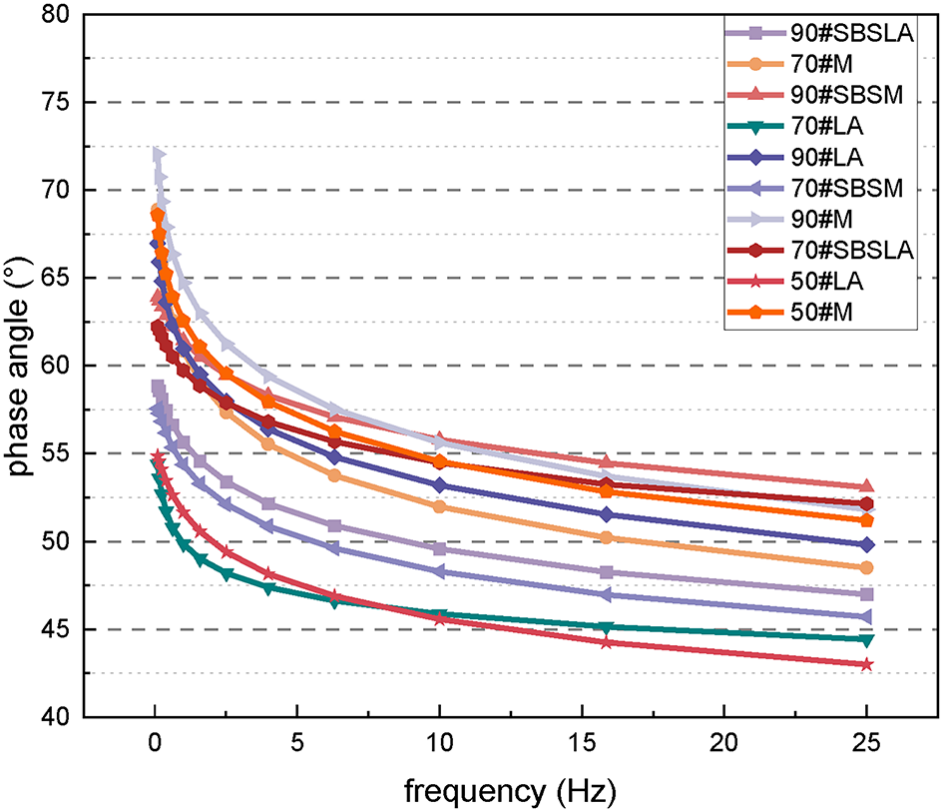
Phase angle curves for different asphalt materials.

Figure 8 shows the complex modulus curves of different asphalt materials. It can be observed that the complex modulus increases with the increase in asphalt grade. Further the addition of the SBS elastomer modifier significantly enhances the complex modulus of the asphalt. For example, the complex modulus of 90# SBS-modified asphalt is greater than that of 90# asphalt. This improvement is attributed to the poor aging resistance of asphalt, where the long-term aging process changes the performance of 90# asphalt, exacerbating the hardening process. Lower-grade materials exhibit reduced sensitivity to loading frequency, resulting in a more stable performance. Furthermore, the addition of the modifier effectively improves the material’s sensitivity to the loading frequency, thereby stabilizing its performance. Compared with the addition of mineral powder, the long-term aging effect is more conducive to stabilizing the complex modulus of asphalt. The phase angle reflects the proportion of viscoelastic components in the material: a smaller phase angle indicates a more elastic material. As shown in Fig. 9, the phase angle increases as the asphalt grade increases. This is because higher-grade asphalt contains fewer elastic components, resulting in a larger phase angle. The SBS elastomer effectively increases the proportion of elastic components in the material. Therefore, the phase angle of SBS-modified asphalt is lower than that of untreated asphalt.

### Relaxation properties of asphalt materials

**Fig. 10:**
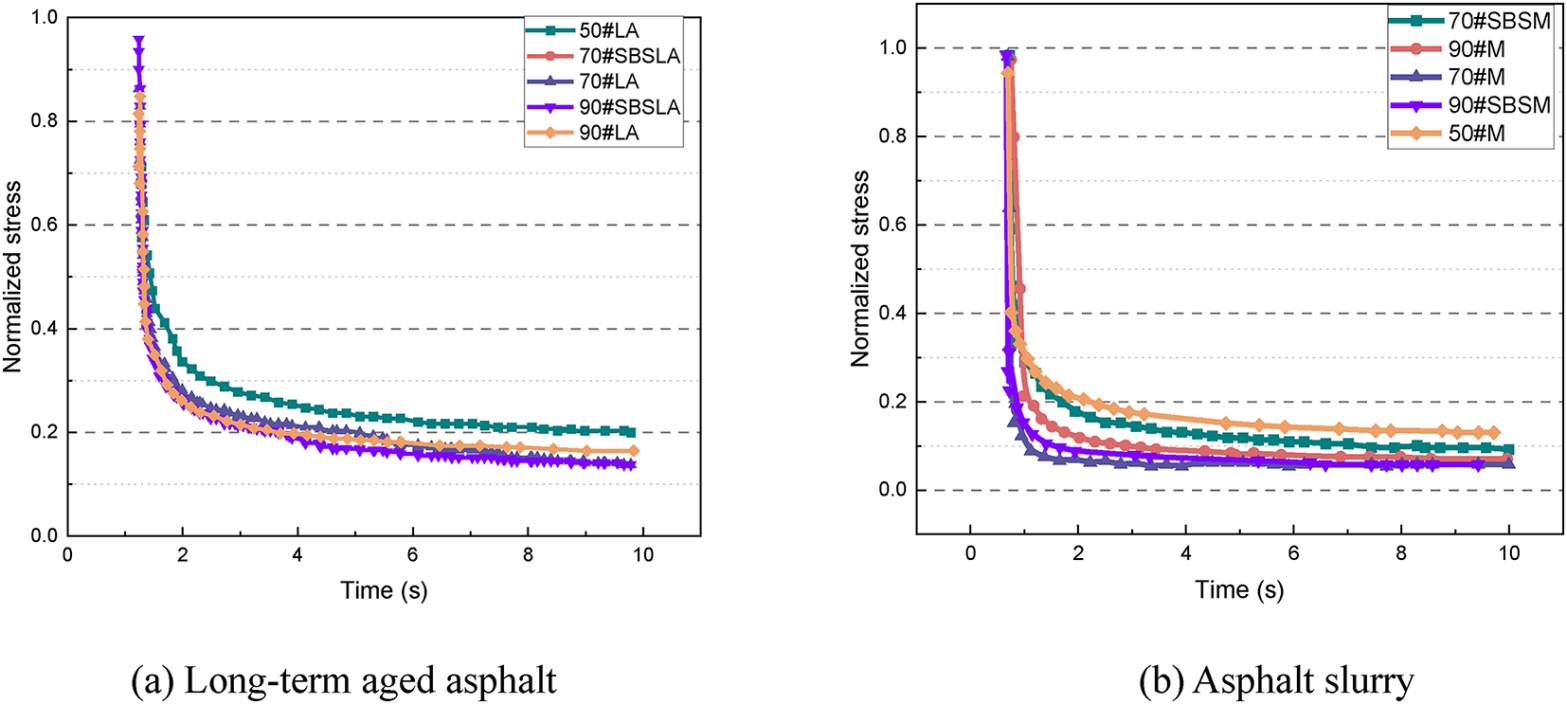
Relaxation curves of various asphalt materials.

Figure 10 presents the relaxation curves of different types of asphalt material during the relaxation stage. As the asphalt grade increases, the relaxation curve becomes steeper, indicating a faster stress relaxation. The addition of the SBS modifier reduces the stress relaxation rate to a certain extent. The relaxation curves of 70#, 90#, and 90# SBS-modified asphalt samples are relatively similar, suggesting that a certain degree of penetration may be insufficient to clearly differentiate the relaxation characteristics of these materials. Additionally, for 90# asphalt, the SBS modifier appears to have limited effectiveness in decelerating its relaxation process.

**Fig. 11:**
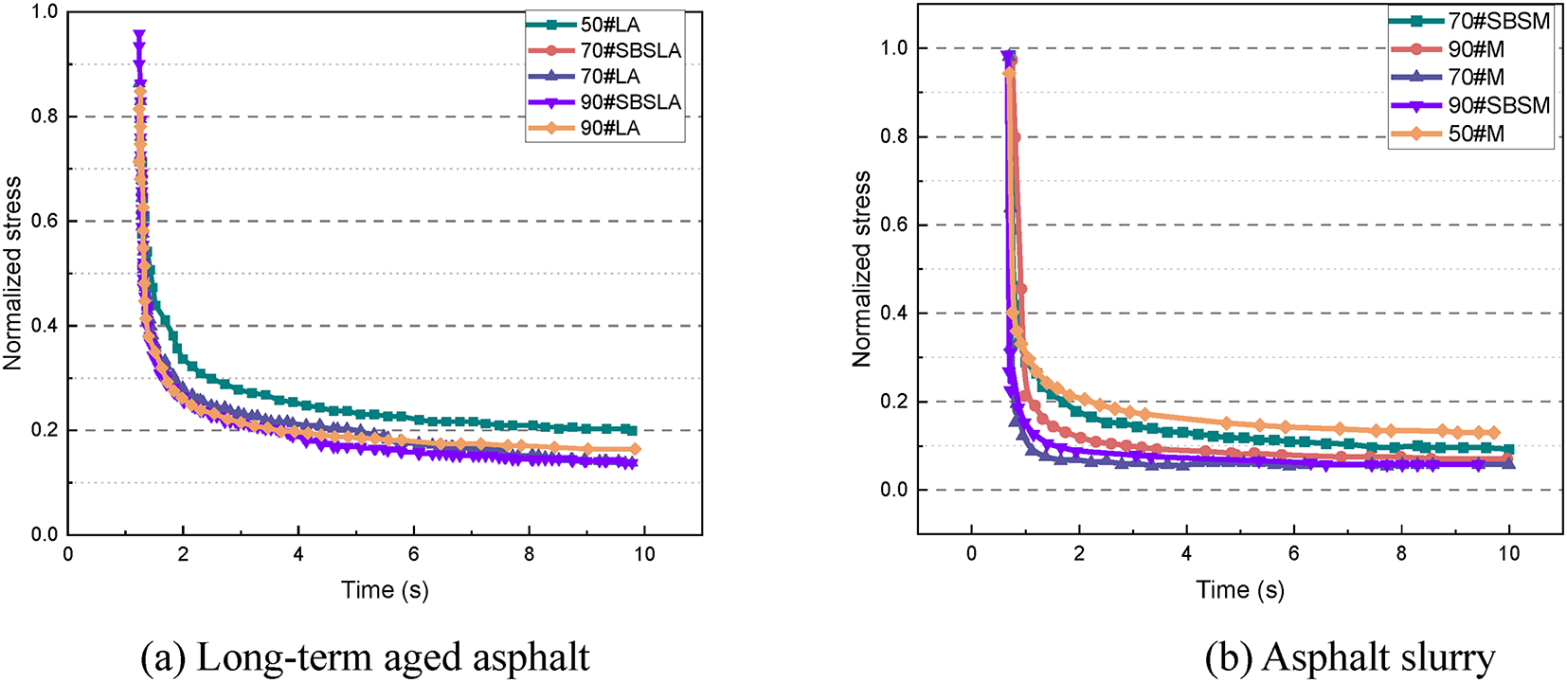
Relaxation curves of various asphalt materials.

Figure 11 shows the strain recovery curves of various asphalt materials during the strain recovery stage. It is evident that the strain recovery curves vary significantly across different asphalt grades. With the increase in the asphalt grade, the curve becomes shallower, indicating a decline in the residual elastic strain recovery ability. This strong correlation between the strain recovery ability and asphalt grade is consistent with the grading principles of material properties based on needle penetration. Additionally, the incorporation of the SBS elastomer effectively improves the strain recovery ability of the asphalt materials. This suggests that the residual elastic strain recovery ability of the material is correlated with its elastic properties to some extent.

### LAS test results

#### Fatigue performance analysis

**Fig. 12:**
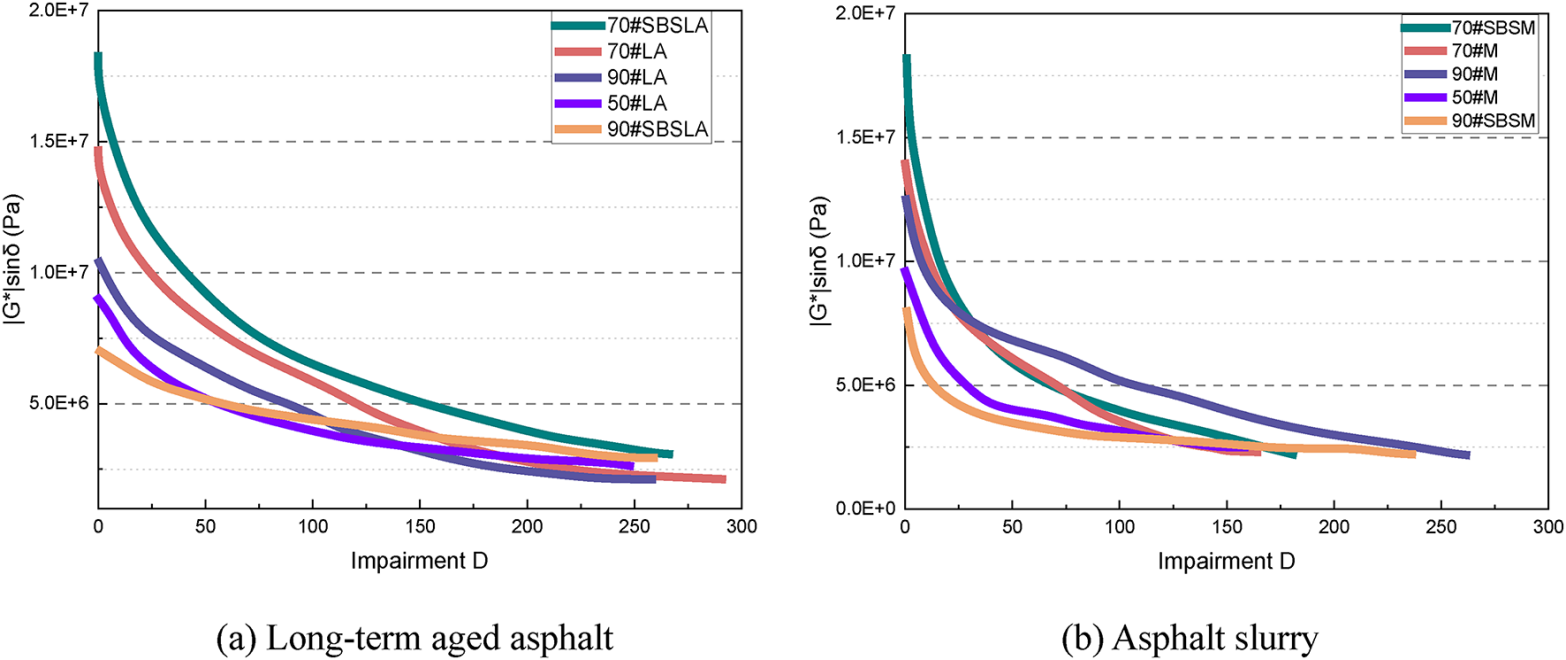
Material damage curve.

The VECD model was employed to analyze the results of the LAS test. Figure 12 presents the damage curve of the material, which can be used to determine its damage limit. Due to its low modulus of elasticity, 50# asphalt and its mastic exhibit a certain level of durability. The addition of the SBS modifier enhances the stability of 70# and 90# aged asphalt and mastic. Although 50# asphalt can withstand loads to a certain extent, the modifier effectively enhances the elastic properties of the material, thereby improving its load stability.

Figure 12 displays the relationship between the fatigue damage and modulus for different asphalt materials. Based on the VECD model, the least squares method was used for curve fitting to derive the parameters required for assessing the fatigue resistance of the material. The corresponding fitting results are presented in Table 4.

**Table 4:** Fitting results for the damage curve of different types of asphalt.

Type of asphalt	C0	C1	C2	α
50#LA	7.34	0.99	0.34	2.68
70#LA	12	0.99	0.43	1.53
70#SBSLA	16.1	2	0.35	1.82
90#LA	7.98	0.49	0.49	1.36
90#SBSLA	5.57	0.4	0.42	1.41
50#M	15.4	4.94	0.2	2.08
70#M	32.3	5.48	0.33	1.29
70#SBSM	43.6	12	0.23	1.61
90#M	28.6	4.53	0.34	1.15
90#SBSM	19.2	3.99	0.26	1.38

**Fig. 13:**
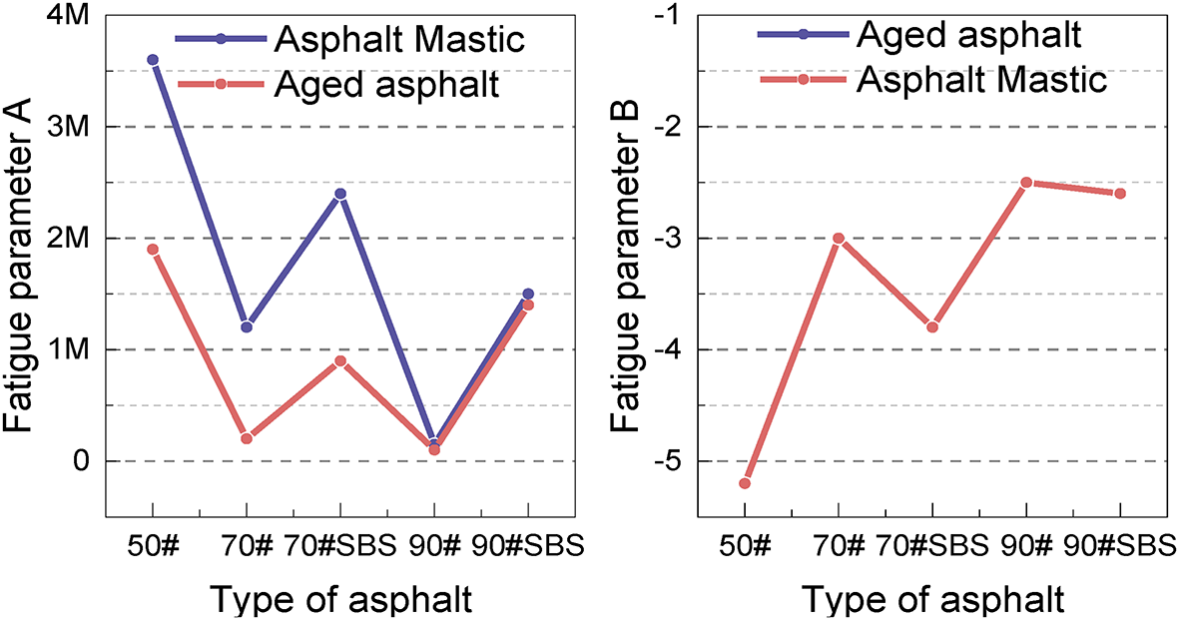
Fatigue parameters of different types of asphalt.

By calculating the parameters A and B of the fatigue prediction equation, the fatigue performance of various asphalt materials can be characterized. Figure 13 presents the fatigue parameter curve for each asphalt type. The fatigue parameter A significantly decreases with the increase in asphalt grade, indicating poorer fatigue performance at higher grades. The fatigue parameter A of modified asphalt is higher than that of the untreated material, indicating that the SBS modifier can effectively improve the fatigue performance under certain conditions. The fatigue parameter A of long-term aged samples is consistently greater than that of asphalt slurry. Conversely, the fatigue parameter B shows an increasing trend with the asphalt grade, indicating that B is more consistent with asphalt penetration. The increase in the value of fatigue parameter B reduces the material’s load sensitivity, resulting in improved durability under complex loading conditions. However, the addition of the modifier results leads to a slight decrease in the fatigue parameter B, which somewhat compromises the load stability of the materials. Furthermore, the fatigue parameter B of asphalt mastic is generally lower than that of aged asphalt, indicating that long-term aging adversely affects the load sensitivity, impairing the material’s ability to adapt to complex loading environments.

**Fig. 14:**
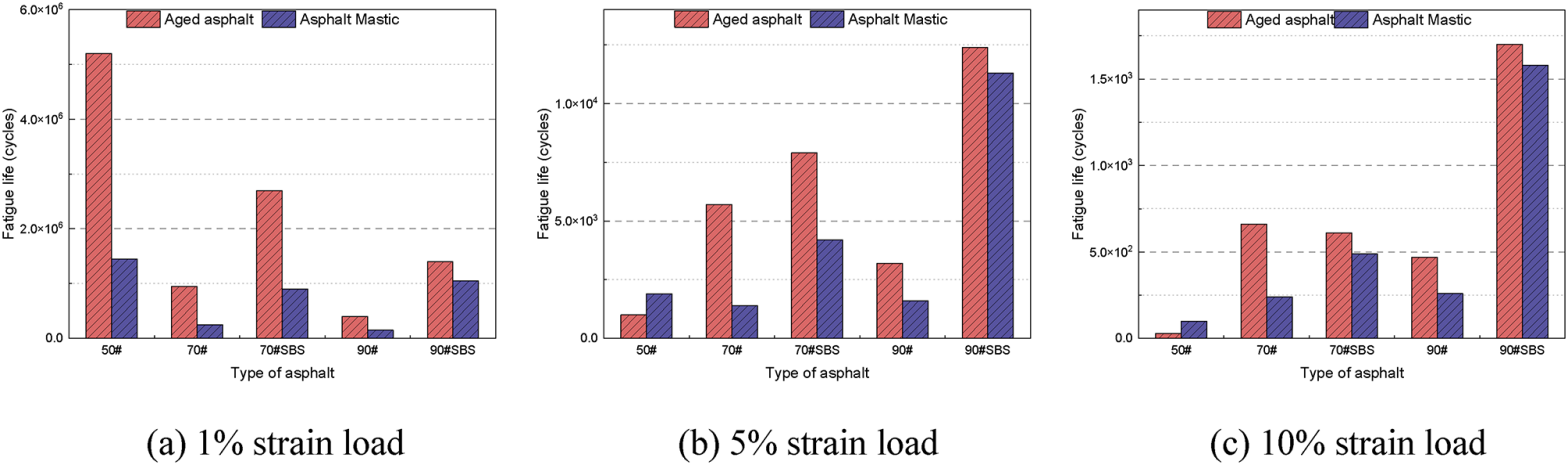
Fatigue life of asphalt at various strain loads.

Figure 14 illustrates the fatigue life of various asphalt materials at different strain loads. At a strain load of 1%, the fatigue life decreases with the increase in asphalt grade for both aged asphalt and asphalt slurry. However, the fatigue life of each material shows a somewhat reversed trend with the increase in strain load. As shown in Fig. 14(b), the fatigue performance of 50# asphalt significantly declines at 5% strain, and all grades of asphalt mastic exhibit similar fatigue lives. Nevertheless, among the aged asphalt samples, 70# asphalt demonstrates the longest fatigue life. As shown in Fig. 14(c), with further increase in the strain amplitude, both aged asphalt and asphalt slurry exhibit the same variation trend, i.e., the fatigue life of 50# asphalt is substantially shorter than that of 70# and 90# asphalt. The variation trends of fatigue life for each grade of asphalt under three fatigue loads indicate that 50# asphalt has the highest load sensitivity, resulting in a more pronounced reduction in fatigue life as the load increases. Under all three loading conditions, the addition of the SBS modifier enhances the fatigue life of asphalt, demonstrating its effectiveness in improving the material’s fatigue resistance.

### Relaxation-based fatigue performance prediction

#### Relationship between the fatigue properties and viscoelasticity

**Fig. 15:**
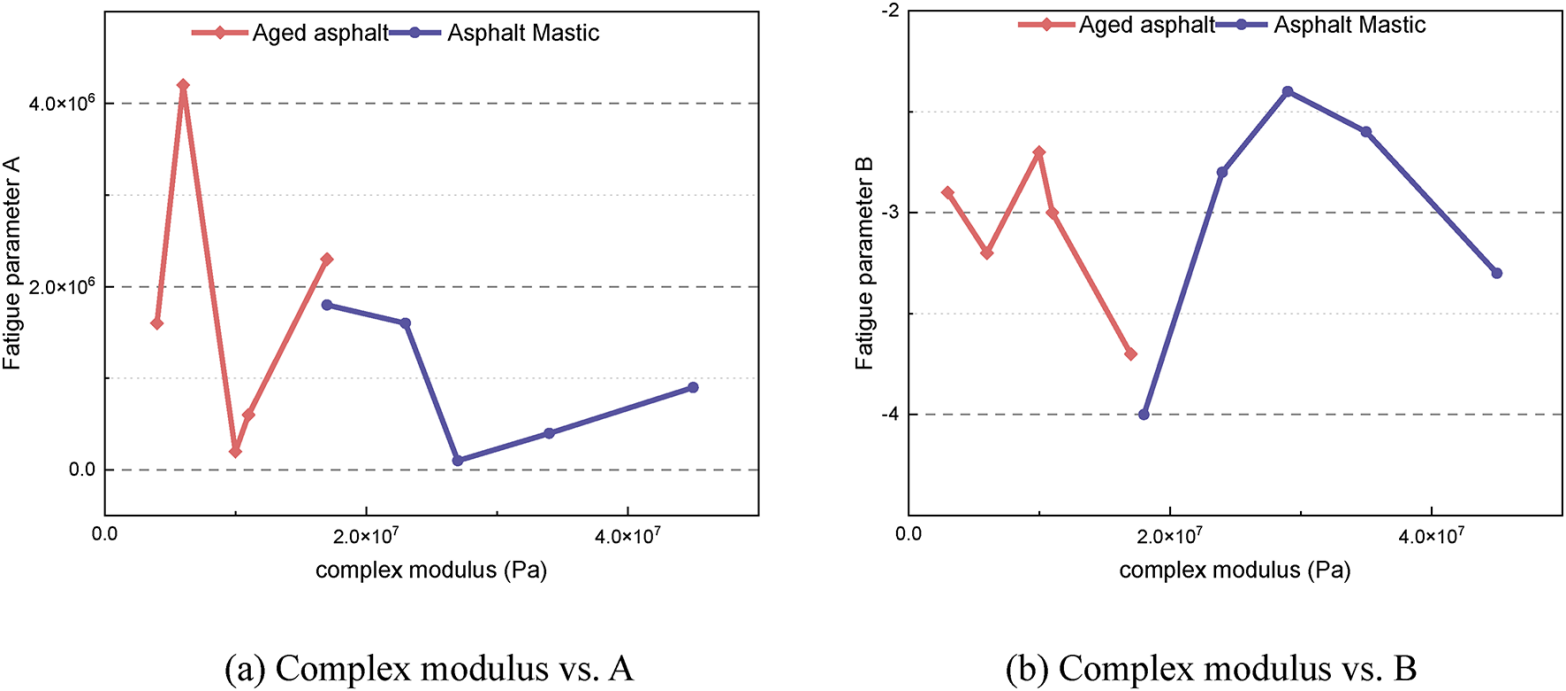
Relationship between the complex modulus and fatigue parameters **A** and **B**.

To analyze the relationship between the fatigue parameters and viscoelastic properties of asphalt, Fig. 15 displays the correlation between the complex modulus and the fatigue parameters for each material. It is clear that for both aged asphalt and asphalt mastic, there is no significant correlation between the complex modulus and the fatigue parameters A and B. Furthermore, as mentioned earlier, the complex modulus is not correlated with the material’s elastic recovery performance. Therefore, the fatigue performance of the material appears to be only weakly related to its modulus.

**Fig. 16:**
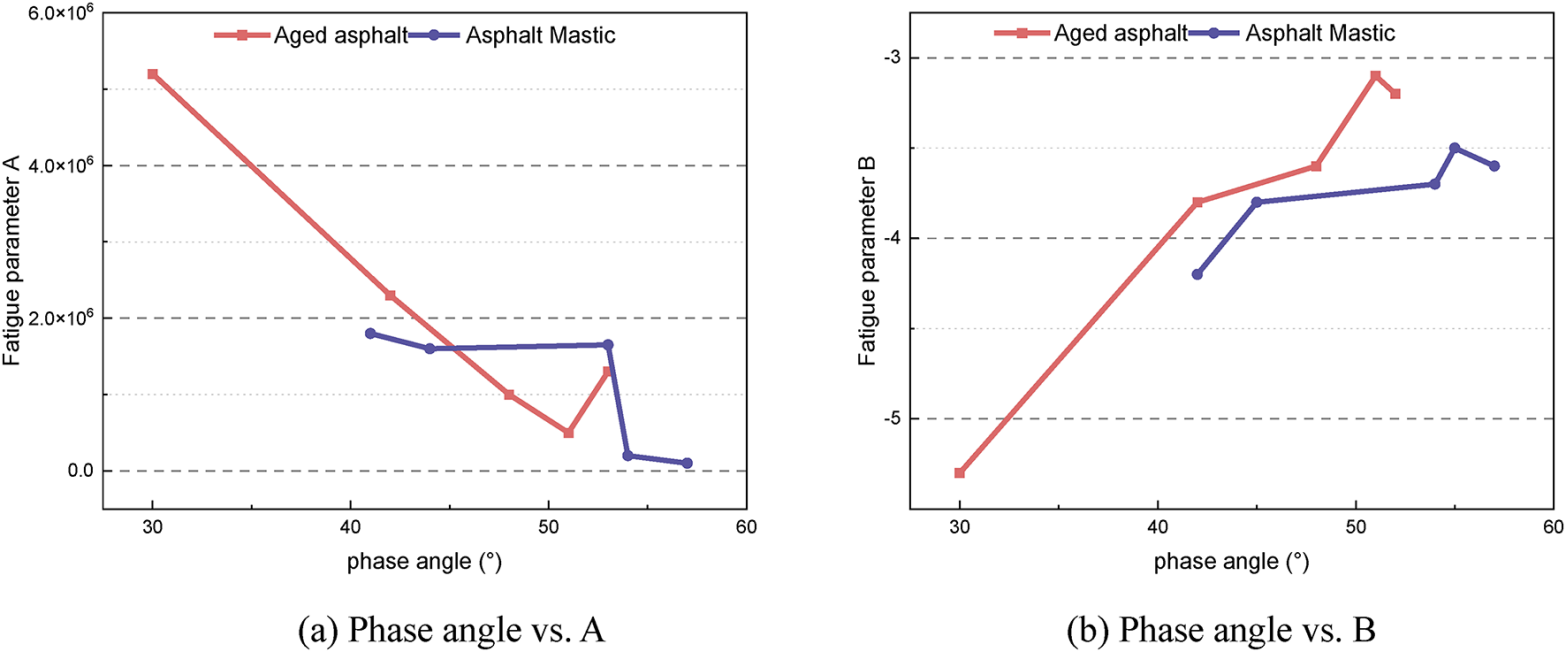
Relationship between the phase angle and fatigue parameters **A** and **B**.

Figure 16 illustrates the relationships between the material’s phase angle and the fatigue parameters. It is evident from Fig. 16(a) that the correlation between the phase angle and fatigue parameter A is weak, indicating that A is not strongly related to the viscoelastic properties of the material. By contrast, Fig. 16(b) reveals a strong correlation between the phase angle and fatigue parameter B. Specifically, the fatigue parameter B increases as the phase angle rises. This suggests that the load sensitivity is related to the proportion of viscous components in the material, and an increase in these components reduces the material’s load sensitivity.

#### Relationship between the fatigue properties, relaxation properties, and other performance indicators

**Fig. 17:**
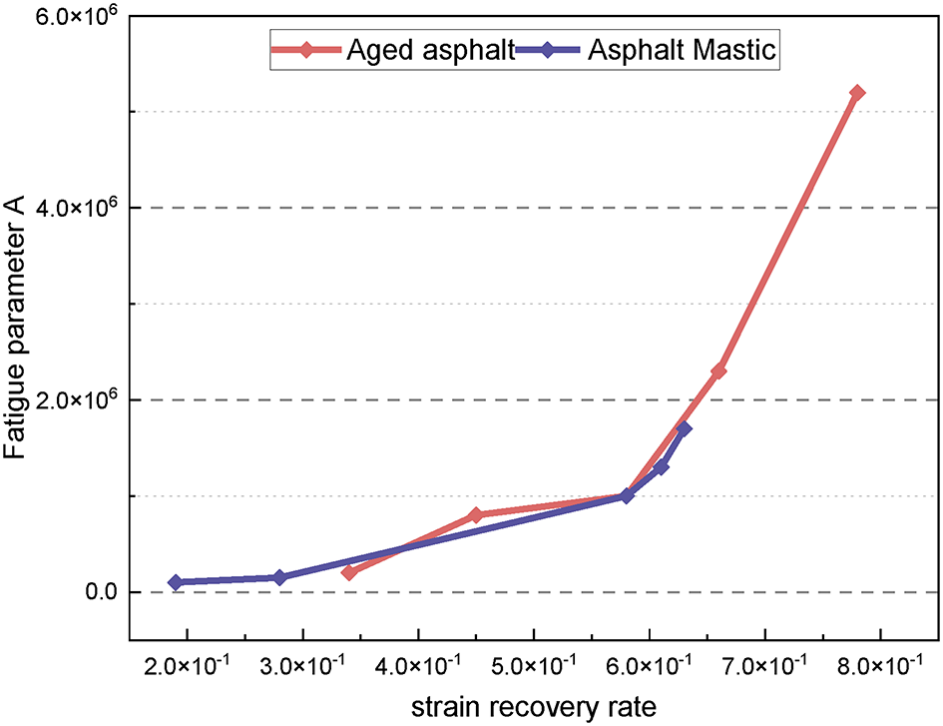
Strain recovery rate vs. fatigue parameter **A** .

**Fig. 18:**
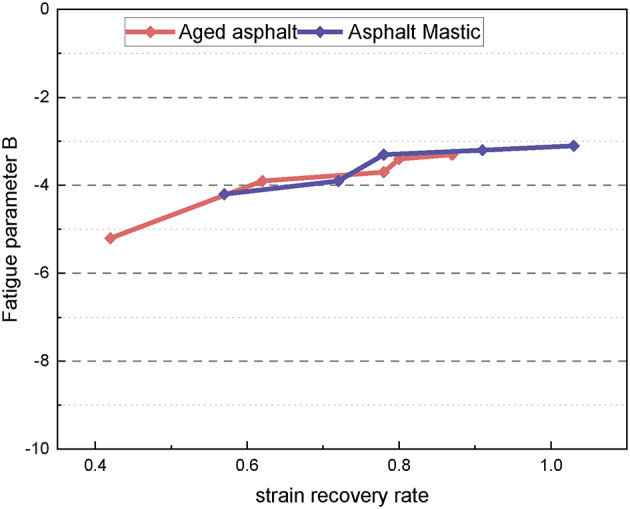
Strain recovery rate vs. fatigue parameter **B**.

The fatigue performance of asphalt material is influenced by its viscous and elastic components. Therefore, the fatigue parameters A and B should be correlated with the material’s viscous and elastic properties. Figure 17 illustrates the relationship between the fatigue parameter A and the strain recovery rate for five types of long-term aged asphalt and five types of asphalt slurry. It is evident that A increases with the increase in the strain recovery rate, demonstrating a strong correlation between them. This suggests that the fatigue parameter A reflects the fatigue impedance, which is positively correlated with the elastic properties of the material. Specifically, higher elastic properties lead to greater fatigue impedance and improved fatigue resistance. Figure 18 shows the relationship between the fatigue parameter B and the strain recovery rate for the same asphalt samples. A reliable positive correlation is observed, indicating that the fatigue parameter B increases as the relaxation rate increases. This implies that a higher relaxation rate reduces load sensitivity, thereby enhancing the material’s durability under complex loading conditions.

#### Fatigue prediction equation

**Fig. 19:**
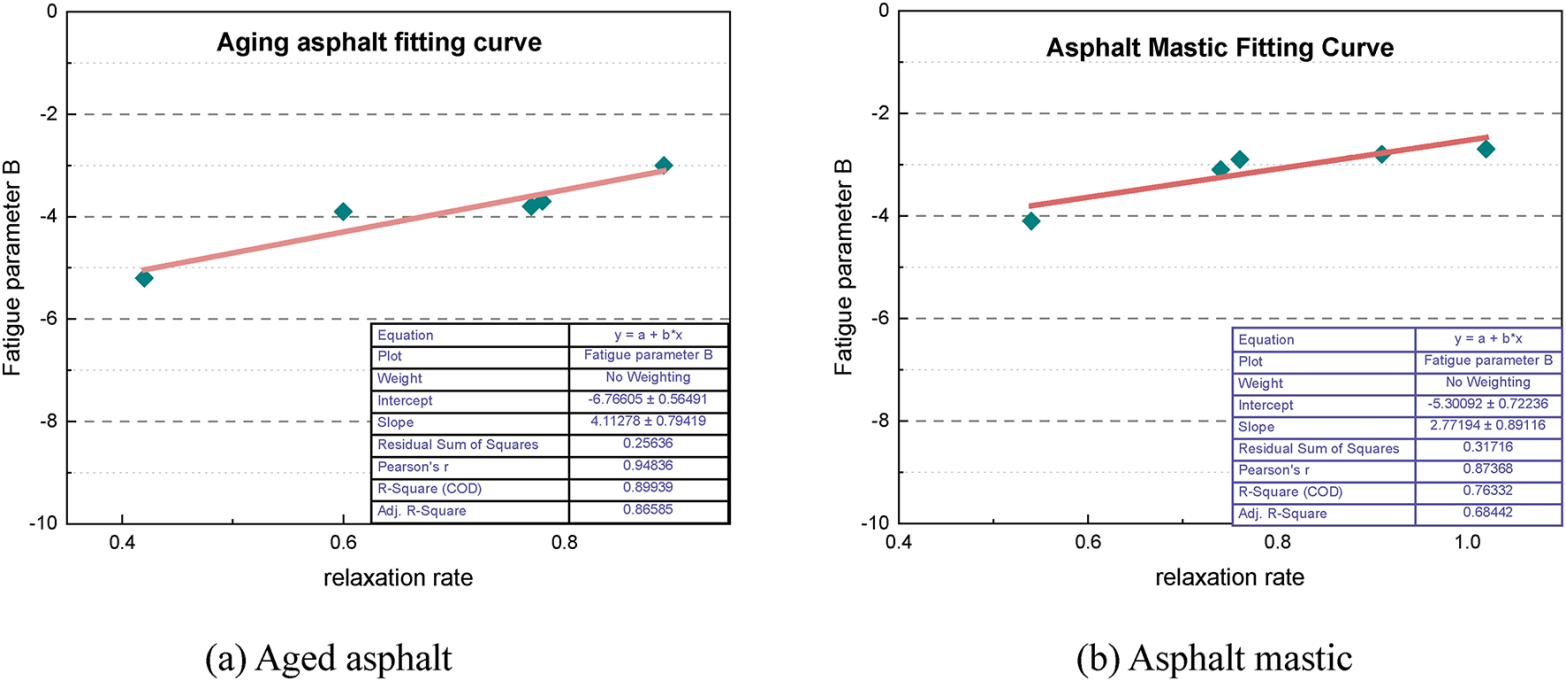
Fitted curves of fatigue parameter B vs. relaxation rate.

Figure 19 presents the fitted curve illustrating the relationship between the fatigue parameter B and the relaxation rate. A strong linear relationship is evident, and the corresponding coefficients of determination (R^2^) for aged asphalt and asphalt mastic are 0.8993 and 0.7633, respectively, indicating a reliable linear fit. This fitting establishes a numerical relationship between the two indicators, which can be used to analyze both the relaxation performance and load sensitivity of the material.

**Fig. 20:**
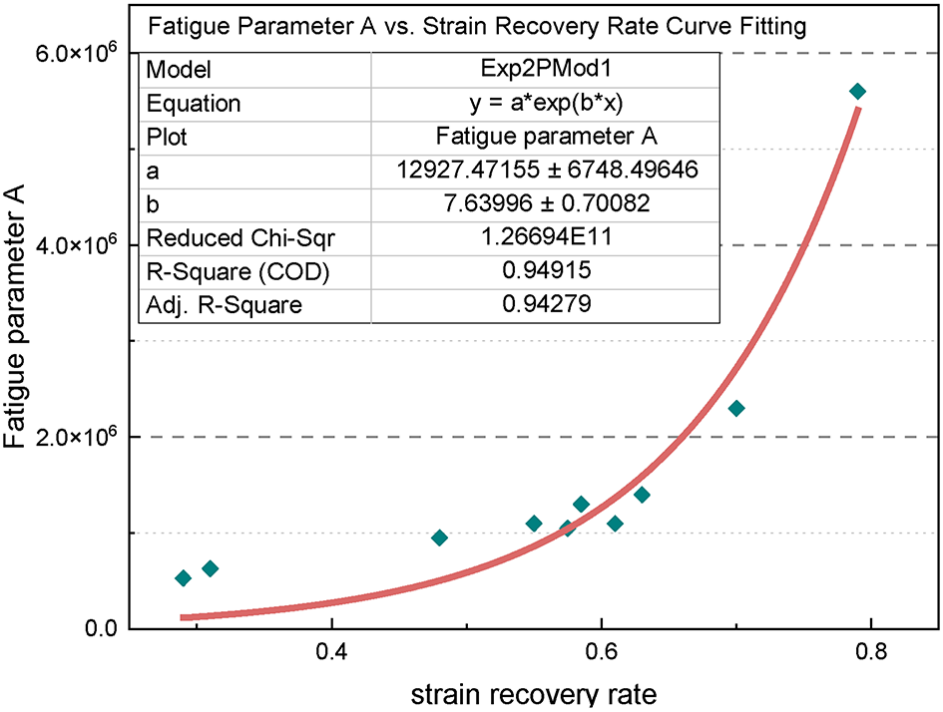
Fitted curve of fatigue parameter **A** vs. strain recovery rate for asphalt material.

A reliable numerical relationship between the rheological properties of the material and the fatigue parameters has been established through fitting. The fatigue parameter A shows a nonlinear increase with the strain recovery rate, which can be modeled using the exponential equation y = ae^bx^. Figure 20 shows the fitted curve for the variation in the fatigue parameter A with the strain recovery rate for the ten asphalt materials. The corresponding coefficient of determination (R^2^) is 0.9931, indicating a high degree of fit. Thus, the numerical relationship between the elastic recovery ability and the fatigue impedance of the materials can be established. The exponential relationship between the fatigue parameter A and the strain recovery rate is expressed as follows:

1 $$\:\text{A}=12927.4715\times\:{\text{e}}^{(7.6399\times\:\text{R})}$$

where R is the strain recovery rate.

**Fig. 21:**
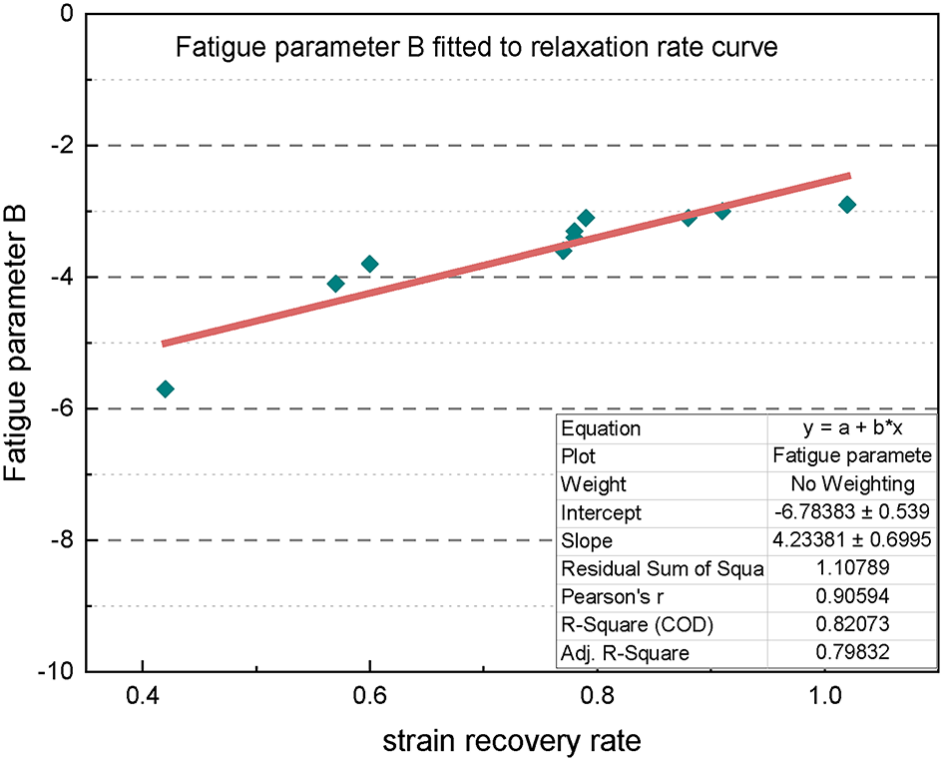
Fitted curves of fatigue parameter **B** vs. relaxation rate for each material.

The fitted curves illustrating the relationship between the fatigue parameter B and the relaxation rate for the ten asphalt materials are presented in Fig. 21, where the coefficient of determination (R^2^) is 0.8183, indicating a very good fit. This confirms a linear relationship between the fatigue parameter B and the material’s relaxation rate, which can be expressed as follows:

2 $$\:\text{B}=-6.7838+4.2338\times\:\text{V}$$

where V is the relaxation rate.

Based on the LAS test results, the fatigue life can be predicted using the following equation:

3 $$\:{\text{N}}_{\text{f}}={\text{A}\left({{\upgamma\:}}_{\text{m}\text{a}\text{x}}\right)}^{\text{B}}$$

where N_f_ represents the fatigue life; A and B are the fatigue parameters; γ_max_ is the peak load strain. The fatigue parameter A serves as an indicator of the material’s fatigue impedance, while the parameter B reflects its degree of load sensitivity. Thus, the fatigue life prediction equation enables an accurate assessment of the material’s fatigue performance.

It is important to note that the viscoelastic properties of asphalt materials are closely related to their fatigue properties. Specifically, the relaxation rate of the material is linearly correlated with its load sensitivity, whereas the strain recovery rate is exponentially related to its fatigue impedance. Therefore, it can be inferred that the fatigue and rheological properties of the material are intrinsically connected. In other words, the fatigue properties can be predicted based on the rheological characteristics. By integrating the functional equations obtained through fitting, the following expression can be derived:

4 $$\:{\text{N}}_{\text{f}}=12927.4715\times\:{\text{e}}^{(7.6399\times\:\text{R}){{{\upgamma\:}}_{\text{m}\text{a}\text{x}}}^{-6.7838+4.2338\times\:\text{V}}}$$

where N_f_ is the fatigue life; R is the strain recovery rate; V is the relaxation rate; γ_max_ is the peak load strain. Equation (4) can be used to predict the fatigue life based on the strain recovery rate R and the relaxation rate V. Both R and V serve as performance evaluation indexes derived from the repeated stress relaxation-recovery test method. Therefore, Eq. (4) is the fatigue performance prediction equation rooted in this testing approach.

## Conclusions

The fatigue performance and relaxation properties of asphalt and its mastic were comprehensively examined using stress relaxation-recovery tests under different conditions. The main results of the study are summarized as follows:

(1) The stress relaxation tests of asphalt binder and its mastic were conducted under different grades, modification conditions, and aging conditions. It was observed that compared with the other parameters, the temperature had a stronger impact on the relaxation properties of the material. In a certain range of strain load, the strain amplitude did not affect the relaxation curve trend of the material.

(2) The rheological property test curve of the asphalt material could be divided into two stages. The first stage was the stress relaxation stage, which could be used to evaluate the relaxation properties of the material. The second stage was the strain recovery stage, which was used to evaluate the residual elastic properties of the material. This test method was proven to be effective in elucidating the relaxation and elastic behaviors of the material under certain conditions.

(3) LAS and repeated stress relaxation-recovery tests were conducted on each material, and the evaluation indexes (relaxation time, relaxation rate, strain recovery rate) of the stress relaxation performance and strain recovery ability were proposed. It was observed that the relaxation performance indexes of the materials did not have a direct relationship with the loss modulus, but they showed a certain correlation with the phase angle of the materials. The relaxation rate and strain recovery rate were identified as reliable evaluation indexes for the relaxation performance and elasticity of asphalt-like materials, respectively, enabling accurate determination of their rheological properties.

(4) The relationship between material damage accumulation and its modulus was established by using the VECD model. The corresponding stress and phase angle versus strain load curves during the material damage process were obtained, leading to the determination of the material’s damage curve.

(5) The load sensitivity of asphalt materials was found to be related to the proportion of viscous components, and more viscous components reduced the load sensitivity of the material. The fatigue parameter A of asphalt material increased with the strain recovery rate, showing a good correlation. The greater the elastic property, the higher the fatigue impedance and the better the fatigue resistance of the material. Additionally, the fatigue parameter B increased with the relaxation rate, indicating that a higher relaxation rate leads to lower load sensitivity. A fatigue performance prediction equation based on the material relaxation test was established using the derived functional relationships. Overall, this study confirmed that the relaxation and fatigue properties of asphalt materials are essentially correlated, and this correlation can be effectively used to evaluate the fatigue properties of the material.

## Data Availability

The datasets used and/or analysed during the current study available from the corresponding author on reasonable request.
